# Evaluation of Implant Stability According to Implant Placement Site and Duration in Elderly Patients: A Prospective Multi-Center Cohort Study

**DOI:** 10.3390/jcm12155087

**Published:** 2023-08-02

**Authors:** Ji-Suk Shim, Moon-Young Kim, Se-Jun An, Eun-Sung Kang, Yu-Sung Choi

**Affiliations:** 1 Department of Dentistry, Korea University Guro Hospital, Seoul 08308, Republic of Korea; 2 Department of Oral and Maxillofacial Surgery, College of Dentistry, Dankook University, Cheonan 31116, Republic of Korea; 3 Department of Prosthodontics, College of Dentistry, Dankook University, Cheonan 31116, Republic of Korea

**Keywords:** implant stability, implant stability quotient, Periotest value, implant stability test, prospective study, elderly patient

## Abstract

The aim of this prospective study is to investigate implant stability and the reliability of different measuring devices according to implant placement site and duration in patients aged over 65 years. The study evaluated 60 implants (diameter: 3.5/4.0/4.5/5.0 mm and length: 8.5/10.0/11.5 mm) in 60 patients aged ≥ 65 years. The implant placement sites were divided into six evenly distributed sections (*n* = 10), i.e., maxillary right-posterior, A; maxillary anterior, B; maxillary left-posterior, C; mandibular right-posterior, D; mandibular anterior, E; mandibular left-posterior, F. Participants visited the hospital six times: implant surgery, 1V; stitch removal, 2V; 1-month follow-up, 3V; 2-month follow-up, 4V; before final restoration delivery, 5V; and after final restoration delivery, 6V. The implant stability was evaluated with the Osstell Mentor (ISQ), Periotest M (PTV), and Anycheck (IST). The mean values of ISQ, PTV, and IST were analyzed (α = 0.05). ISQ, PTV, and IST results of 4V and 5V were significantly higher than those of 1V (*p* < 0.05). The lowest ISQ results occurred in the E location at 4V and 5V (*p* < 0.05). In all mandibular locations, IST results of 6V were significantly higher than those of 1V, 2V, 3V, and 4V (*p* < 0.05). ISQ results were negatively correlated with PTV and positively correlated with IST, and PTV was negatively correlated with IST. By considering various factors affecting the stability of the implant, it is necessary to determine the appropriate implant load application time. This could help increase the implant success rate in elderly patients. And as a diagnostic device for implant stability and the evaluation of osseointegration in elderly patients, Anycheck was also able to prove its relative reliability compared to Osstell ISQ Mentor and Periotest M.

## 1. Introduction

As the elderly population shows increased life expectancy without a longer frailty period, medical care aiming to maintain the quality of life for elderly patients is increasing [1]. For similar reasons, implant treatment for elderly patients is increasing worldwide. A marked increase in the number of implant surgeries after the year 2000 for patients aged over 70 years has been reported by the University of Bern [1]. The number of patients aged over 75 years with an implant-supported prosthesis has increased in Switzerland over the past 20 years [2]. Recently, due to developments in implant surfaces, a procedure that allows application of a functional load faster than the traditional osseointegration period has been introduced in many studies. Since the differentiation rate of osteoblasts decreases with increasing age, older patients should be given a longer period for osseointegration [3,4,5,6].

In addition, clinical data on the implant osseointegration period according to the volume and density of the alveolar bone, and whether bone is transplanted, are also needed. It is of utmost importance to quantify implant stability at various time points and to determine and establish the timing of loading [7,8]. The changes in implant stability over time during the healing period could reveal more definite results upon comparing the physiological responses following implant placement in young and elderly patients. Implant stability occurs in two stages: primary and secondary [7,8]. Primary stability at implant installation is achieved by physical congruence between the surgically created bone bed and the implant, which is dependent on the macroscopic implant design, surgical technique, and bone density [7]. During the osseointegration healing period, bone gradually forms inside the implant threads, and thus, secondary stability is attained by an incremental degree of bone-to-implant contact [7]. The transition period in which the primary stability decreases while the secondary stability becomes established is a critical period with increased risk of osseointegration following micro-movement of the implant [7]. Primary and secondary implant stability significantly relies on factors such as bone density, wound healing process, and bone metabolism, which could be affected by the age of the patient [8].

Elderly patients undergoing implant surgery possess a higher risk compared to younger patients, owing to their medical condition and the adverse effects of medications being taken. Moreover, the differences in the bone quality and metabolism between elderly and young patients could result in differences in the osseointegration patterns following implant placement. The bone matrix is composed of bone mineral, collagen, bone water, and non-collagenous proteins [3], and the components of the bone matrix change with aging, ultimately causing deterioration of the mechanical properties of bone, including increased brittleness and skeletal fragility [4,5]. In particular, the changes in non-collagenous proteins, which have molecular signaling functions regulating bone remodeling, alter the ability of bone to respond to external stimuli [3,6].

Despite age-related risk factors, previous studies did not demonstrate definite results that advanced age negatively affects osseointegration. Brocard et al. reported the lowest success rate in older patients [9]. Bertl et al. reported that patients over 80 years of age showed a higher rate of early implant loss than younger patients [10]. However, Schimmel et al. reported high implant survival rates in patients aged over 75 years (97.3% for 1 year and 96.1% for 3 years) based on a systematic review and meta-analysis [11]. Jemt et al. demonstrated that younger patients, rather than older edentulous patients, had a higher risk of implant failure [12]. As implant failure is a multifactorial complication, previous studies demonstrated that implant failure can be affected by the compliance method of the patient group and the selected statistical analysis methods based on age.

Therefore, the measurement of implant stability is even more necessary now, and two types of widely used non-invasive diagnostic methods have been developed and examined: resonance frequency analysis (RFA) and damping capacity analysis (DCA) [13,14]. In RFA, a method used by the Osstell ISQ Mentor (Osstell, Göteborg, Sweden), the stiffness of the implant–bone interface is outputted from the resonance frequency that is the response to oscillations applied to the implant–bone system [14]. One DCA system device, Periotest M (Siemens AG, Bensheim, Germany), has been utilized to assess the mobility of natural teeth and is maintained to have the potential to reliably evaluate the stability of the implant–bone interface [13,14]. Recently, the new DCA system device Anycheck (Neobiotech, Seoul, Republic of Korea) has been released; it is a measuring device that uses percussion and has improved accuracy and reduced patient discomfort by reducing the intensity of the percussion [15]. This system evaluates the duration of contact between an impacting rod and a healing abutment. It strikes the healing abutment six times over 2 s and converts the duration into implant stability test (IST) values [15]. This system strikes a healing abutment with less force than Periotest M does and has a function that allows it to stop automatically if the stability is low to protect the implant [15]. However, little is known about the factors affecting the IST values or the reliability of the device.

Multiple previous studies have reported on the correlation between RFA and DCA device results indicating the stability of the same implant. An in vitro study presented the strong correlation between the results from RFA and DCA devices [16]. However, an in vivo study presented the relatively lower correlation between the results from RFA and DCA devices [17]. The different results under experimental and clinical conditions suggest that there are clinical factors which affect the reliability of implant stability measuring devices. In the experimental condition, implant stability can be examined without any obstacles and the device can be positioned in relation to the implant in an ideal way. Clinically, examination of the stability of the implant in the oral cavity may have access difficulties due to the cheek, tongue, and contralateral teeth. These obstacles may unfavorably influence the factors needed for accurate measurement of implant stability, including exact contact between implant and device, the angle of the device to the implant, and the angle of the device against gravity. In addition, in the clinical condition, the accessibility and angle of the device are influenced by the location of the implant in the oral cavity (anterior/posterior, left/right, and mandible/maxilla).

Therefore, reliable implant stability measurement is required to evaluate the degree of osseointegration according to the implant placement site. However, there are few prospective clinical studies evaluating implant stability and measuring device reliability, according to the implant placement site and post-implantation duration in older patients. Therefore, the aim of this in vivo study was to evaluate implant stability and the reliability of different measuring devices, according to the implant placement site in the oral cavity and the duration of implantation in patients over 65 years. Additionally, one RFA device (Osstell) and two DCA devices (Periotest and Anycheck) were used to examine implants located in the maxillary right-posterior, maxillary anterior, maxillary left-posterior, mandibular right-posterior, mandibular anterior, and mandibular left-posterior positions.

The null hypotheses were that (1) the implant placement site and duration of implantation in the oral cavity do not affect implant stability and measuring device reliability in patients over 65 years, and (2) a correlation of 1 is shown by the three measuring devices.

## 2. Materials and Methods

### 2.1. Study Design

This study was performed with 60 patients: 30 men and 30 women aged 65 and over needing implant treatment at Korea University Guro Hospital and Dankook University Dental Hospital from 2020 to 2022. This prospective study was registered at the public clinical trials database before the commencement of the study (Clinical research information service of National Research Institute of Health in Republic of Korea, KCT0005721). Clinical trials were conducted at two dental centers following approval by the local medical ethics boards (2020GR0580 and DKUDH 2020-11-001), and the methods were conducted according to the relevant guidelines [15,16,17]. If a tooth needed to be extracted first, it was extracted according to the delayed implant criteria, and bone healing was allowed for 3–4 months prior to implant placement. If implant placement failed or if a participant withdrew their consent, they were considered dropouts. The sample size calculation was based on previous studies comparing implant stability diagnostic devices, and the result (correlation coefficiency = 0.777) was used as the standard [18]. Using a sample number calculation program (G power ver 3.1; Heinrich-Heine-Universität, Düsseldorf, Germany), the sample size calculation was based on the results of a paired t-test (α = 0.05, β = 0.8, two-tailed).

### 2.2. Patient Selection

**Figure 1:**
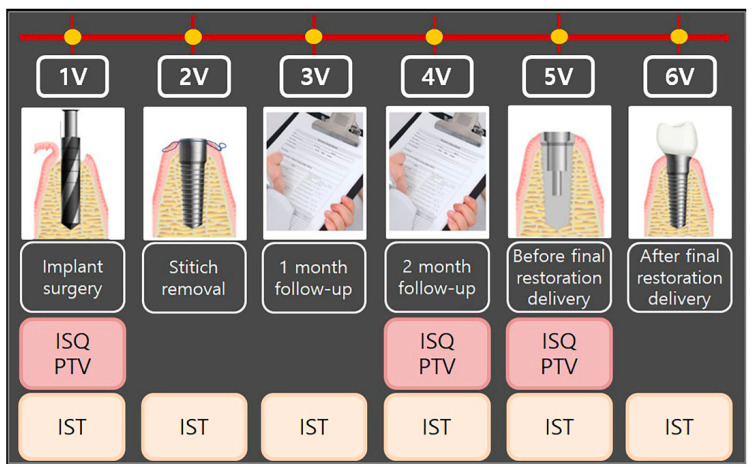
Flowchart of the study process. 1V, first visit; 2V, second visit; 3V, third visit; 4V, fourth visit; 5V, fifth visit; 6V, sixth visit. ISQ, implant stability quotient; PTV, Periotest value; IST, implant stability tester value.

All patients had read, understood, and signed the written informed consent form at least 7 days before implant surgery. Once written consent was obtained, clinical staff verified that the participant satisfied several inclusion and exclusion criteria for participation (Table 1). No patients were excluded. Participants visited the hospital six times, including at implant surgery (1V), stitch removal (2V), 1-month follow-up (3V), 2-month follow-up (4V), before final restoration delivery (3- to 4-month follow-up) (5V), and after final restoration delivery (3- to 4-month follow-up) (6V). At 1V, 4V, and 5V, implant stability was evaluated with the Osstell ISQ Mentor and Periotest M, and at 1V, 2V, 3V, 4V, 5V, and 6V, stability was evaluated with the Anycheck. The study process is shown in Table 2 and Figure 1.

### 2.3. Surgical Procedure

**Figure 2:**
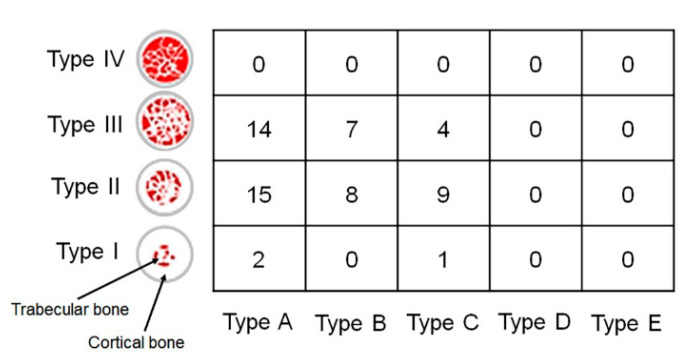
The bone quality around the implant analyzed by cone-beam computed tomography according to Lekholm and Zarb classification. Type I, the entire bone is composed of very thick cortical bone; Type II, thick layer of cortical bone surrounds a core of dense trabecular bone; Type III, thin layer of cortical bone surrounds a core of trabecular bone of good strength; and Type IV, very thin layer of cortical bone with low-density trabecular bone of poor strength. Type A, most of the alveolar ridge is present; Type B, moderate residual ridge resorption has occurred; Type C, advanced residual ridge resorption has occurred, and only basal bone remains; Type D, some resorption of basal bone has started; Type E, extreme resorption of the basal bone has taken place. Number, Number of implants placed in the site.

The implants were evenly distributed in the oral cavity (Table 3). The implants were placed in the maxillary right-posterior (A), maxillary anterior (B), maxillary left-posterior (C), mandibular right-posterior (D), mandibular anterior (E), and mandibular left-posterior (F) positions. An appropriate implant was selected and placed by evaluating bone quality and quantity after dental cone-beam computed tomography (CBCT) and fabricating a stent for implantation at the appropriate site for each patient. A bone-level tapered implant (CMI IS-II, Neobiotech, Seoul, Republic of Korea) with a sandblasted, large grit, acid-etched surface and internal hex connection was used in the present study. The characteristics of the implants (diameter: 3.5/4.0/4.5/5.0 mm and length: 8.5/10.0/11.5 mm) used in this study are presented in Table 4. To evaluate implant stability following the determined timetable, all the implants were placed in the non-submerged state. All the implants were placed without implementing any bone augmentation procedure. The drilling process followed the manufacturer’s instruction to drill the site first with a point Lindemann drill, followed by surgical drills. To achieve similar insertion torque values (ITV) of 35 Ncm between implants, a well-trained researcher carefully drilled each implant bed at a regular depth and angle in each hospital. ITV was measured at 20 rpm and 8 Hz to a maximum of 35 Ncm using a drilling unit specially designed for implant surgery (iCTmortor, WH-1, Dentium, Seoul, Republic of Korea). All the implants were placed only with the handpiece of the drilling unit. CBCT was used immediately after surgery to evaluate the bone quality around the implant [19]. The bone quality types are characterized according to Lekholm and Zarb classification (Figure 2).

### 2.4. Measurement of Implant Stability

A well-trained, right-handed researcher measured the implant stability in each hospital. To prevent the fixation force of the implant from changing during the process of installing and releasing the healing abutment, implant stability was evaluated with the Osstell ISQ Mentor first. For measurement with the Osstell ISQ Mentor, the smart peg was connected manually to the implant fixture. All the devices were used in accordance with the manufacturer’s instructions. The manufacturer of the Osstell ISQ Mentor recommends that the device tip be held close (2.0–4.0 mm) to the top of the smart peg without touching it and at approximately 45° to the smart peg top. Since the accuracy of the measurement may be improved if the same implant is measured repeatedly in succession, the implants in each area were measured in turns. After the measurements with the Osstell ISQ Mentor, healing abutments (Neobiotech, Seoul, Republic of Korea) were connected to the implant with 30 Ncm torque, using a torque ratchet, by selecting a height that could expose about 2.0 mm from the gingival level. After healing abutment connection, implant stability was measured with Anycheck and Periotest M. The percussion of Periotest M was carried out perpendicularly to the longitudinal axis of the abutment, holding the handpiece parallel to the floor. An angulation of more than 11 degrees from the horizontal plane is registered by the device as an error [13]. The start button was kept on top, and the rod and the healing abutment surface were required to maintain a distance of 0.6–2.0 mm. The metal rod of Anycheck and the long axis of the implant were put perpendicular to each other, similar to Periotest M. The tip of the tapping rod was in slight contact with the healing abutment. The device maintained the contact angle between 0 and 30 degrees. For standardization of measurement after the prosthesis was installed, the IST of the prosthesis was measured at a position 5.0 mm in the long axis direction from the terminal upper line of the fixture analog in the working model using digimatic vernier calipers (Mitutoyo Co., Kanagawa, Japan). All device results were measured in the buccal (or labial) direction and were recorded by one examiner in each hospital. Anycheck strikes a healing abutment six times over 3 s, while Periotest M is accelerated toward the implant tooth sixteen times in 4 s. Osstell ISQ Mentor, Periotest M, and Anycheck measurements were conducted three times for inserted fixtures.

### 2.5. Statistical Analysis

This study was performed to evaluate the accuracy and reliability of IST based on the ISQ and PTV values that have proven accuracy for differences between groups according to the post-implantation duration and the location of the implant. All the data were evaluated using SPSS Statistics for Windows v25.0 (IBM SPSS Inc., Chicago, IL, USA). One-sample Kolmogorov–Smirnov tests were performed to analyze the normality of the collected data and, based on the normality of the test results, statistical analysis was performed. One-way analysis of variance (ANOVA) tests were used to compare differences between the groups according to the implantation location, followed by Tukey’s post hoc comparisons. Based on the stability evaluation value at the time of implant placement, the increase in stability until final prosthesis insertion was repeatedly measured, and the mean and standard deviation calculations were based on each implant and analyzed by Friedman tests (α = 0.05). Pearson’s correlation test was used to evaluate the correlations between ISQ and PTV, between ISQ and IST, and between PTV and IST. Correlation coefficients (r) are evaluated as very strong (0.80 ≤ r ≤ 1.00), strong (0.60 ≤ r ≤ 0.79), moderate (0.40 ≤ r ≤ 0.59), weak (0.20 ≤ r ≤ 0.39), very weak (0.00 < r ≤ 0.19), and no correlation (r = 0) for both positive and negative values [20,21]. A two-way ANOVA was performed to compare the correlations among the location of the implants in the oral cavity (anterior/posterior, left/right, mandibular/maxillary). A Tukey’s honest significant difference test was performed as the post hoc test, with the significance level set at 95% (α = 0.05).

## 3. Results

**Figure 3:**
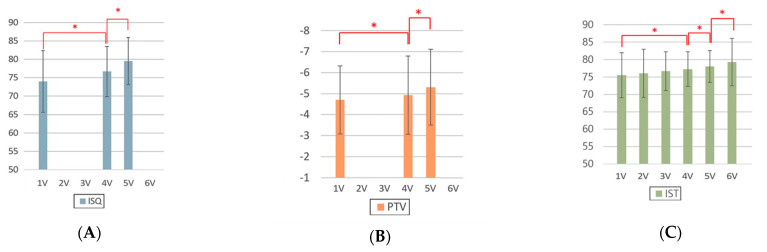
Analysis of implant stability measurements made with different devices according to the duration. (**A**) ISQ, (**B**) PTV, and (**C**) IST. ISQ, implant stability quotient; PTV, Periotest value; IST, implant stability tester value. 1V, first visit; 2V, second visit; 3V, third visit; 4V, fourth visit; 5V, fifth visit; 6V, sixth visit. * denotes a significant difference, with *p* < 0.05.

Implant stability measurement was performed using various devices. Mean values and standard deviations of ISQ, PTV, and IST among the groups, according to post-implantation duration, are shown in Figure 3. For all the ISQ, PTV, and IST results, the implant stability results at the 2-month follow-up and before the final restoration delivery were significantly higher than those at the time of implant surgery. The significant differences in ISQ, PTV, and IST among the groups, according to post-implantation duration, are shown in Table 5. For the ISQ and PTV, there were statistically significant differences between the first and fourth visits, first and fifth visits, and fourth and fifth visits (*p* < 0.05). For the IST, there were significant differences at all points except those between the first and second visits, first and third visits, second and third visits, and third and fourth visits (*p* < 0.05).

**Figure 4:**
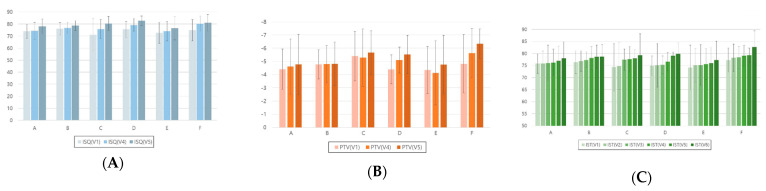
Analysis of implant stability measurements made with different devices according to dental implant placement site and duration. (**A**) ISQ, (**B**) PTV, and (**C**) IST. ISQ, implant stability quotient; PTV, Periotest value; IST, implant stability tester value. A, maxillary right-posterior; B, maxillary anterior; C, maxillary left-posterior; D, mandibular right-posterior; E, mandibular anterior; F, mandibular left-posterior. 1V, first visit; 2V, second visit; 3V, third visit; 4V, fourth visit; 5V, fifth visit; 6V, sixth visit.

Mean values and standard deviations of implant stability measurements made with different devices according to dental implant placement site and duration are shown in Figure 4. For the ISQ, there were statistically significant differences according to the post-implantation duration for each location of the inserted implants between the first and fourth visits, first and fifth visits, and fourth and fifth visits (Table 6).

The results of Pearson’s correlation between the mean ISQ, mean PTV, and mean IST results are presented in Table 7. At the first visit, the r between the ISQ and PTV results was −0.208, verifying the weak negative correlation (*p* = 0.049). The r between the ISQ and IST results was 0.567, verifying the moderate positive correlation (*p* < 0.001). Additionally, the r between the PTV and IST results was −0.490, verifying the moderate negative correlation (*p* < 0.001). At the fourth visit, the r between the ISQ and PTV results was −0.298, verifying the weak negative correlation (*p* = 0.001). The r between the ISQ and IST results was 0.367, verifying the weak positive correlation (*p* = 0.003). Additionally, the r between the PTV and IST results was −0.701, verifying the strong negative correlation (*p* < 0.001). At the fifth visit, the r between the ISQ and PTV results was −0.252, verifying the weak negative correlation (*p* = 0.005). The r between the ISQ and IST results was 0.503, verifying the moderate positive correlation (*p* < 0.001). Additionally, the r between the PTV and IST results was −0.479, demonstrating the moderate negative correlation (*p* < 0.001).

The results of all groups for the implant stability values between the locations of implants and the positions of the arch in Osstell ISQ Mentor, Periotest M, and Anycheck are shown in Table 8. The ISQ results showed statistically significant differences at the fourth (*p =* 0.016) and fifth visits (*p =* 0.042). In the PTV results, there were no significant differences in the correlations between the locations of the implants at all the visits (*p* > 0.05). In the IST results, there was a statistically significant difference only at the fifth visit (*p =* 0.044) in the correlations between the locations of implants.

## 4. Discussion

In the present study, the accuracy of implant stability measurement devices was evaluated under clinical conditions affecting the reliability of the devices. In addition to the results from the devices, the valid impacts and the angle formed by the handpiece with the horizontal plane were measured to analyze the reasons for inaccurate results. The results showed that the implant placement site and the post-implantation duration in the oral cavity affected implant stability and the reliability of the measuring devices in patients over 65 years of age.

Devices for evaluating implant stability are either RFA or DCA and each device has distinct characteristics depending on their operating principles. A DCA device is convenient for measurement without an additional process if a healing abutment is installed; more factors during measuring should be controlled to derive accurate results compared to RFA devices [22]. According to previous studies, the PTV is influenced by the length of the fixture and the healing abutment, the position and direction of percussion, and the angle of the handpiece [23,24].

Between the DCA devices, Periotest M was significantly affected by the position of artificial bone model impact error, while Anycheck showed consistently low impact error in this study. The results showed that Anycheck was able to provide a relatively stable measurement under unfavorable access conditions. Anycheck measured while in contact with the implant, the device does not move minutely during measurement, and it is possible to measure stably at the desired position. However, Periotest M is unstable because it is measured at a certain distance from the implant. Faulkner et al. reported that Periotest M was very sensitive to the angulation of the handpiece and to the position at which the Periotest M impacted the abutment [25]. A small change in the angle of the handpiece from 90 degrees to the abutment may cause a PTV difference between 2.5 and 4.0, as the rod hits an inconsistent point of abutment [26,27]. In addition, the variation in PTV was approximately 1.5 or 1 to 2 PTV depending on the height of the striking point per millimeter [25,26].

Some studies have investigated conflicting results for both RFA and DCA systems. Lee et al. investigated the strong correlation (0.981) between the ISQ and IST in an in vitro study [13], while a systematic review showed a weak correlation (−0.294) between the ISQ and PTV [15]. Additionally, the correlating ISQ and PTV readings of the buccal surface during implant installation were moderately negatively statistically significantly correlated (−0.466) between the two types of device for all 80 patients in the randomized clinical trial by Andreotti et al. [14]. In this study, there were weak negative statistically significant correlations: −0.208 at 1V, −0.298 at 4V, and −0.252 at 5V, between ISQ and PTV. There were moderate positive statistically significant correlations: 0.567 at 1V, 0.367 at 4V, and 0.503 at 5V, between ISQ and IST. The results of this study are similar to the reported correlations in previous studies [14,15]. A factor that can affect the results is when using the DCA device clinically, the examiner is limited by patient cooperation, space, and access, unlike the laboratory study, where standardized models for measurement permit certain conditions. Thus, in vivo analyses have additional sources of error that could result in reduced accuracy of measurement. The results presented the weak or moderate statistically significant correlation between the three measuring devices.

Several studies have presented the strong correlation between ISQ and PTV, while others have presented no correlation [28,29,30,31]. Because of the discrepancies, standardized implant stability values have not yet been proved and analyses have been performed by other analytic methods, such as the measurement of ITV and radiographic and clinical examinations. There were moderate to high negative statistically significant correlations: −0.490 at 1V, −0.701 at 4V, and −0.479 at 5V, between PTV and IST. There was a moderate positive statistically significant correlation coefficient of 0.414 between the 5V and 6V, in IST (*p* < 0.001).

Some studies have investigated whether both the Osstell ISQ and Periotest devices could reliably evaluate the stability of the implant [13,29,32]. Lachmann et al. maintained that both the Osstell ISQ and Periotest presented acceptable reliability in expecting the implant stability in an in vitro study [13]. Also, Pang et al. reported the strong correlation between the ISQ and PTV post-surgery and 2 months later [29]. An animal study showed the strong correlation between ISQ and PTV [31]. Additionally, some studies demonstrated that although both the Osstell ISQ and Periotest devices were useful for analyzing the stability of the implant, the Osstell ISQ was more accurate than the Periotest systems, presenting high reliability [33,34]. However, some studies have found conflicting results for both the Osstell ISQ and Periotest systems [14,31]. Considering the controversy, both the Osstell ISQ and Periotest were evaluated with the Anycheck in this study [31].

There are well-known inconveniences and limitations of the Osstell ISQ and Periotest systems. The Osstell ISQ is a non-invasive system that can evaluate implant stability based on the structural analysis principle [35]. This system could be fairly reliable when the bone–implant interface is rigid and the implants have achieved osseointegration. However, when the implant–bone interface is doubtful or is not rigid, the ISQ results tend to change [36,37]. Additionally, use of the Osstell ISQ requires removal of the upper fixture component and the smart-peg connection when evaluating implant stability and this could cause limitations and inconvenience. Long-term study on Periotest has shown that it could objectively measure implant stability [38,39]. However, some studies have reported that the devices lack sensitivity [40,41]. This is because the Periotest, designed for natural dentition, evaluates a wide dynamic range. However, the range used for evaluating implant stability is limited [30]. Other studies have shown that an even narrower range of −4 to −2 or −4 to +2 is required for clinically osseointegrated implants [42,43]. Moreover, PTV was unable to identify implants with borderline stability or those in the process of osseointegration [44]. PTVs have also been criticized for vulnerability to operator variables and lack of resolution [45,46]. The IST results were consistent with ISQ results. Additionally, the IST results range from 1 to 99. Usage of the Anycheck does not require unscrewing of the healing abutment and the procedure is therefore easier than that of the Osstell ISQ.

This clinical study investigated the reliability of each device by comparing RFA and DCA devices with different measurement principles. The results presented the effect of the implant placement site and the post-implantation duration in elderly patients on the reliability of each measuring device. In addition, an attempt was made to accurately obtain the angle the handpiece would make with the ground and the number of effective strokes when measuring stability with Anycheck.

Furthermore, Lombardi et al. demonstrated that early marginal bone remodeling was significantly influenced by implant insertion depth and factors related to biological width establishment, and reported that deep implant insertion, thin peri-implant mucosa, and short abutments were associated with greater marginal bone loss up to 6 months after prosthetic loading [47]. Fu et al. reported that implant diameter had a more profound impact than length on ITV/primary ISQ, and bone density played a considerable role in ITV/primary ISQ determination [48]. Bone density and ITV may have a greater effect than primary ISQ on marginal bone loss [48]. All the sites of missing teeth were included for implant placement in this study. However, the bone density and volume of the alveolar bone, which may affect implant stability, differ based on the location of bone [7]. Therefore, further investigation with implants placed in confined locations is necessary.

However, the limitation of this in vivo study was that the reliability of Anycheck was based on the correlations with the other systems and the agreement rate of each device was not evaluated in this study. Additionally, the design of the study could not compare the systems with the implant osseointegration and further large-scale in vivo studies are needed for clinical use. Additional studies are also needed to ascertain the reliability of the Anycheck system through analysis of the patient’s face shape, mouth size, and 3D structure of the oral cavity. In addition, the factors that may affect the measured values, such as implant length, implant diameters, bone quality, bone density, soft tissue, patient opening, and saliva, cannot be excluded during clinical use of this prospective multi-center study. Therefore, further in vivo studies are required to estimate the accuracy and accessibility of the devices in clinical use.

## 5. Conclusions

Within the limitations of our study, it is necessary to determine the appropriate implant load application time by considering various factors affecting the stability of the implant. This could help increase the implant success rate in elderly patients. And as a diagnostic device for implant stability for evaluation of osseointegration in elderly patients, Anycheck was also able to prove its relative reliability compared to Osstell ISQ Mentor and Periotest M.

## Figures and Tables

**Table 1:** The list of inclusion and exclusion criteria for participation in this study.

Criteria	Lists
Inclusion	(1) Those requiring implant placement
	(2) The anatomical conditions under which non-submerged implant placement was favorable at stage I surgery
	(3) Those who agreed to participate in the clinical study and signed the consent form for the clinical study
Exclusion	(1) The heavy smokers (>10 cigarettes/day)
	(2) The presence of bone defects requiring bone augmentation
	(3) Those requiring implant placement following implant failure
	(4) Those with uncontrolled medical condition
	(5) Those confirming or suspecting psychological problems

**Table 2:** Summary of the study process.

Visit	Observation Period	Observation and Clinical Examination Items
1V	Implant surgery	Measurement of implant stability by all the devices
2V	Stitch removal	Measurement of implant stability by Anycheck
3V	1-month follow-up	Measurement of implant stability by Anycheck
4V	2-month follow-up	Measurement of implant stability by all the devices
5V	Before final restoration delivery (3- to 4-month follow-up)	Measurement of implant stability by all the devices
6V	After final restoration delivery (3- to 4-month follow-up)	Measurement of implant stability by Anycheck

1V, first visit; 2V, second visit; 3V, third visit; 4V, fourth visit; 5V, fifth visit; 6V, sixth visit.

**Table 3:** Implant placement locations.

Location of Implant Placement	Abbreviation	Number
Maxillary right-posterior	A	10 (Male: 5, Female: 5)
Maxillary anterior	B	10 (Male: 5, Female: 5)
Maxillary left-posterior	C	10 (Male: 5, Female: 5)
Mandibular right-posterior	D	10 (Male: 5, Female: 5)
Mandibular anterior	E	10 (Male: 5, Female: 5)
Mandibular left-posterior	F	10 (Male: 5, Female: 5)

**Table 4:** The characteristics of the implants used in this study.

Characteristics	Size (mm)	Number
Length	8.5	21
	10.0	35
	11.5	4
Diameter	3.5	3
	4.0	20
	4.5	17
	5.0	20

**Table 5:** Statistical analysis of implant stability measurements made with different devices according to the duration.

Value	1V:2V	1V:3V	1V:4V	1V:5V	1V:6V	2V:3V	2V:4V	2V:5V	2V:6V	3V:4V	3V:5V	3V:6V	4V:5V	4V:6V	5V:6V
ISQ	-	-	0.001 *	<0.001 *	-	-	-	-	-	-	-	-	<0.001 *	-	-
PTV	-	-	0.049 *	<0.001 *	-	-	-	-	-	-	-	-	0.001 *	-	-
IST	0.488	0.096	0.012 *	<0.001 *	<0.001 *	0.215	0.048 *	0.005 *	<0.001 *	0.127	0.007 *	<0.001 *	0.043 *	<0.001 *	0.032 *

ISQ, implant stability quotient; PTV, Periotest value; IST, implant stability tester value. 1V, first visit; 2V, second visit; 3V, third visit; 4V, fourth visit; 5V, fifth visit; 6V, sixth visit. The *p*-values of Visit I–II were calculated as Friedman test results between the mean Visit I values and the mean Visit II values (*p* < 0.05). The significant differences (*p*-value) in the ISQ, PTV, and IST values among the groups according to the post-implantation duration. * denotes a significant difference, with *p* < 0.05.

**Table 6:** Statistical analysis of implant stability measurements made with different devices according to dental implant placement site and duration.

Location	Value	1V:2V	1V:3V	1V:4V	1V:5V	1V:6V	2V:3V	2V:4V	2V:5V	2V:6V	3V:4V	3V:5V	3V:6V	4V:5V	4V:6V	5V:6V
A	ISQ	-	-	0.728	0.002 *	-	-	-	-	-	-	-	-	0.003 *	-	-
	PTV	-	-	0.596	0.305	-	-	-	-	-	-	-	-	0.719	-	-
	IST	0.937	0.857	0.744	0.499	0.172	0.910	0.788	0.583	0.249	0.883	0.594	0.093	0.485	0.267	0.503
B	ISQ	-	-	0.703	0.149	-	-	-	-	-	-	-	-	0.046 *	-	-
	PTV	-	-	0.961	0.932	-	-	-	-	-	-	-	-	0.934	-	-
	IST	0.500	0.334	0.139	0.071	0.176	0.507	0.196	0.080	0.147	0.072	0.018	0.150	0.268	0.600	0.954
C	ISQ	-	-	0.078	0.002 *	-	-	-	-	-	-	-	-	0.001 *	-	-
	PTV	-	-	0.847	0.654	-	-	-	-	-	-	-	-	0.136	-	-
	IST	0.882	0.136	0.185	0.104	0.141	0.059	0.174	0.176	0.181	0.906	0.704	0.498	0.772	0.451	0.529
D	ISQ	-	-	0.031 *	<0.001 *	-	-	-	-	-	-	-	-	0.009 *	-	-
	PTV	-	-	0.006 *	<0.001 *	-	-	-	-	-	-	-	-	0.086	-	-
	IST	0.903	0.474	0.023 *	<0.001 *	0.001 *	0.929	0.449	0.056	0.048 *	0.085	<0.001 *	0.001 *	<0.001 *	0.002 *	0.458
E	ISQ	-	-	0.561	0.159	-	-	-	-	-	-	-	-	0.152	-	-
	PTV	-	-	0.615	0.329	-	-	-	-	-	-	-	-	0.005 *	-	-
	IST	0.729	0.743	0.612	0.383	0.048 *	0.902	0.676	0.619	0.046 *	0.718	0.635	0.012 *	0.729	0.017 *	0.434
F	ISQ	-	-	0.047 *	0.019 *	-	-	-	-	-	-	-	-	0.669	-	-
	PTV	-	-	0.005 *	<0.001 *	-	-	-	-	-	-	-	-	0.021 *	-	-
	IST	0.454	0.177	0.073	0.109	0.007 *	0.885	0.564	0.333	0.049 *	0.266	0.408	0.023 *	0.815	0.033 *	0.052

A, maxillary right-posterior; B, maxillary anterior; C, maxillary left-posterior; D, mandibular right-posterior; E, mandibular anterior; F, mandibular left-posterior. ISQ, implant stability quotient; PTV, Periotest value; IST, implant stability tester value. 1V, first visit; 2V, second visit; 3V, third visit; 4V, fourth visit; 5V, fifth visit; 6V, sixth visit. The *p*-values of Visits 1–2 were calculated as Friedman test results between the mean Visit 1 values and the mean Visit 2 values (*p* < 0.05). * denotes a significant difference, with *p* < 0.05.

**Table 7:** Results of Pearson’s correlation between the mean ISQ, PTV, and IST values.

Value	1V			4V			5V		
	Correlation Coefficient	Size of Correlation	*p*-Value	Correlation Coefficient	Size of Correlation	*p*-Value	Correlation coefficient	Size of Correlation	*p*-Value
ISQ-PTV	−0.208	weak	0.049 *	-0.298	weak	0.001 *	−0.252	weak	0.005 *
ISQ-IST	0.567	moderate	<0.001 *	0.367	weak	0.003 *	0.503	moderate	<0.001 *
PTV-IST	−0.490	moderate	<0.001 *	-0.701	strong	<0.001 *	−0.479	moderate	<0.001 *

ISQ, implant stability quotient; PTV, Periotest value; IST, implant stability tester value. 1V, first visit; 2V, second visit; 3V, third visit; 4V, fourth visit; 5V, fifth visit; 6V, sixth visit. * denotes a significant difference, with *p* < 0.05.

**Table 8:** Results of the two-way ANOVA of all groups for the implant stability values between the locations of the implants and the positions of arch with the mean ISQ, PTV, and IST values.

Value		1V	2V	3V	4V	5V	6V
ISQ	Location	0.561	-	-	0.198	0.048 *	-
	Arch	0.655	-	-	0.062	0.362	-
	Location × Arch	0.134	-	-	0.016 *	0.042 *	-
PTV	Location	0.119	-	-	0.052	0.007 *	-
	Arch	0.250	-	-	0.870	0.157	-
	Location × Arch	0.720	-	-	0.321	0.520	-
IST	Location	0.923	0.809	0.162	0.185	0.436	0.137
	Arch	0.977	0.811	0.516	0.752	0.747	0.289
	Location × Arch	0.186	0.199	0.472	0.170	0.044 *	0.259

ISQ, implant stability quotient; PTV, Periotest value; IST, implant stability tester value. * denotes a significant difference, with *p* < 0.05.

## Data Availability

The data that support the findings of this study are available from the corresponding author upon reasonable.
